# Vitamin D Receptor Polymorphisms and Immunological Effects of Vitamin D in Hashimoto’s Thyroiditis

**DOI:** 10.3390/ijms262110576

**Published:** 2025-10-30

**Authors:** Mateusz Pakosiński, Martyna Żyła, Anna Kamieniak, Natalia Kluz, Paulina Gil-Kulik

**Affiliations:** 1 Student Scientific Society of Clinical Genetics, Medical University of Lublin, 11 Radziwillowska Str., 20-080 Lublin, Poland; matio930@wp.pl (M.P.); martyna.zyla38@gmail.com (M.Ż.); akamieniak28@gmail.com (A.K.); 2 Department of Clinical Genetics, Medical University of Lublin, 11 Radziwillowska Str., 20-080 Lublin, Poland; nataliakluz99@gmail.com; 3 Doctoral School, Medical University of Lublin, 20-093 Lublin, Poland

**Keywords:** Hashimoto, vitamin D receptor, polymorphism, antibodies, supplementation, deficiency, mRNA

## Abstract

Introduction: Vitamin D is involved in numerous processes and is obtained both exogenously and endogenously. Its active form is 1,25-dihydroxycholecalciferol, which exerts its biological effects via the vitamin D receptor (VDR). The main factors influencing VDR density are polymorphisms of the *VDR* gene, which may affect, e.g., gene mRNA stability and also *VDR* gene expression. There are four main polymorphic sites within the gene, BsmI, ApaI, FokI and TaqI, and two polymorphisms related to the gene promoter: GATA and Cdx2. One of the functions of vitamin D is to modulate the immune system. It affects T lymphocytes, B lymphocytes and dendritic cells. Currently, vitamin D deficiency is a common global problem that is associated with an increased risk of autoimmune diseases, including Hashimoto’s thyroiditis. Numerous studies have demonstrated an association between low vitamin D levels and elevated thyroid-stimulating hormone (TSH) levels, and have also proven the existence of a negative correlation between vitamin D levels andanti-thyroid peroxidase (anti-TPO) and anti-thyroglobulin (anti-Tg) antibody titers. Review objectives and a concise summary of the methodology: The review aims to analyze studies examining the relationship between specific *VDR* polymorphisms, vitamin D levels, and the development of various diseases, with a particular emphasis on Hashimoto’s thyroiditis. This review is based on original and review articles written in English published between March 2018–November 2024 searched primarily in the PubMed, and additionally in Google Scholar databases. A narrative review of the literature was conducted. Conclusions: The presence of specific *VDR* polymorphisms influences the effectiveness of vitamin D supplementation, but the role of supplementation in the prevention of autoimmune diseases has not been definitively confirmed. To date, studies have primarily involved relatively small groups of patients with significant population heterogeneity, with case–control investigations being the most common. Therefore, further research on larger, more homogeneous groups is recommended to achieve more standardized results. Additionally, the influence of epigenetic factors modulating VDR activity and its interactions with the environmental factors is also important.

## 1. Introduction

### 1.1. The Scale of the Problem

Currently, Hashimoto’s thyroiditis is one of the most common thyroid diseases. It is also known as lymphocytic thyroiditis, which is characterized by a diffuse lymphocytic infiltration and elevated levels of antibodies, such as those against thyroglobulin (anti-Tg) and thyroid peroxidase (anti-TPO). Current investigation indicates that this disease is mediated by both environmental and genetic factors [1,2]. The incidence of this disease is steadily increasing, which has been confirmed, especially over the last 30 years. It predominantly affects women, with an estimated ratio of 0.3 to 1.5 cases per every 1000 people in the general population, and the risk of developing it increases with age [3]. Hashimoto’s thyroiditis is currently the leading cause of primary hypothyroidism diagnosed in both adults and adolescents [2,3]. Vitamin D deficiency is also a globally widespread phenomenon, and it is notably evident in Poland [4]. A two-stage study conducted by Chlebna-Sokół et al. examined 720 children aged 9–13 years and found that 84.2% had a 25-hydroxyvitamin D (25(OH)D) level below 50 nmol/L, whereas only 15.8% participants had levels within the normal range. These findings confirm the previously mentioned thesis that vitamin D deficiency is very common in the Polish population [5]. The vast majority of published studies on the impact of vitamin D deficiency on health risks demonstrate objective evidence of positive conclusions [4]. The available literature describes findings that may indicate a link between vitamin D deficiency and poor cancer prognosis [6]. This association is particularly evident in lung cancer and lymphoma. Patients with sufficient vitamin D levels (≥62 nmol/L) had less than half the risk of mortality compared to those with reduced 25(OH)D levels (<46 nmol/L). Furthermore, an increase in 25(OH)D levels between subsequent measurements was associated with a significantly lower risk of cancer-related death [7].

Research shows that there is a correlation between vitamin D levels and the presence of antibodies specific to Hashimoto’s thyroiditis, but a precisely defined relationship has not been established so far. This indicates that the number of studies on this issue is insufficient, and the topic is not fully explored.

The aim of this study is: (1) to investigate the influence of vitamin D receptors density in patients with Hashimoto’s thyroiditis, (2) to examine the relationship between vitamin D levels and the incidence of various diseases, with a particular emphasis on Hashimoto’s thyroiditis, and (3) to discuss how vitamin D affects the immune system in the context of Hashimoto’s thyroiditis.

### 1.2. Vitamin D—Its Synthesis and Functions

Vitamin D is a fat-soluble vitamin existing in two main forms: in the form of vitamin D2 (ergocalciferol), which is of plant origin, and in the form of vitamin D3 (cholecalciferol), which is synthesized in human skin. Both forms are biologically inactive and require enzymatic conversion to their active metabolites [8]. Upon exposure to ultraviolet B (UVB) radiation, 7-dehydrocholesterol in the skin absorbs UVB and is converted to previtamin D3, a thermodynamically unstable compound that spontaneously isomerizes to vitamin D3 (cholecalciferol) within hours [9]. After ingestion or endogenous synthesis, vitamin D undergoes its first phase of metabolism, hydroxylation in the liver, which produces 25(OH)D, which is commonly used as a biomarker of vitamin D levels in the blood. Subsequently, 25(OH)D is transported to the kidneys, where it undergoes further hydroxylation, contributing to the formation of 1,25-dihydroxyvitamin D (1,25(OH)2D), the biologically active form of vitamin D. Its biological functions are mediated via the vitamin D receptor (VDR) [10]. The hepatic hydroxylation step is not regulated and depends solely on the availability of circulating previtamin D3. In contrast, renal hydroxylation is strictly regulated and catalyzed by 1-alpha-hydroxylase, encoded by CYP27B1, which is primarily expressed in the proximal tubule, where most calcitriol production occurs. Extrarenal expression of CYP27B1 has also been identified. However, it primarily mediates the local action of vitamin D, exerting negligible effects on plasma calcitriol levels. Increased CYP27B1 expression correlates with enhanced calcitriol production, which proves that transcription is the dominant mechanism controlling calcitriol synthesis [11]. Vitamin D is transported to target tissues primarily by vitamin D-binding protein (DBP), which is characterized by a high affinity for binding to vitamin D and its metabolites. This allows for the maintenance of proper vitamin D levels in the blood.

In contrast, albumins exhibit low affinity for vitamin D but enable its transport, especially when DBP levels are low. Vitamin D bound to DBP demonstrates greater bioavailability [12].

In addition to its synthesis inside the human body, this vitamin can be obtained exogenously. Dietary sources of vitamin D2 include mushrooms and yeast, whereas vitamin D3 is found in cod liver oil, fatty fish and egg yolks. However, since it is difficult to meet the daily requirement through diet, daily supplementation is recommended [13,14,15,16].

The fundamental function of vitamin D is to maintain normal calcium levels and participate in bone remodeling and mineralization by influencing calcium-phosphate metabolism [10]. In the intestines, the active form of vitamin D increases the absorption of calcium and phosphorus, while in the kidneys, it increases the reabsorption of calcium and reduces phosphate reabsorption. Another significant function of vitamin D is its involvement in immune processes [10,12].

Several studies have also shown an association relationship between low vitamin D levels and an increased risk of cardiovascular diseases, cancers, depression, and schizophrenia, but that causal roles remain unproven in many cases. These diseases have been discussed in detail in studies and reviews [8,9,10,12,13,14]. However, there is currently insufficient evidence to justify the effectiveness of vitamin D supplementation for the prevention or treatment [8,12].

### 1.3. Vitamin D Receptor mRNA Expression and Its Impact on the Development of Autoimmune Diseases

VDR polymorphisms may affect VDR mRNA expression and cytokine secretion. Both of these factors play a significant role in the pathogenesis of autoimmune thyroid diseases, such as Graves’ disease and Hashimoto’s thyroiditis. The VDR, as a nuclear transcription factor activated by 1,25-dihydroxyvitamin D_3_, regulates the expression of numerous genes involved in immune modulation, cell proliferation, and apoptosis. Dysregulation of this pathway may therefore alter immune tolerance and contribute to the loss of self-recognition, which is a hallmark of autoimmune thyroid disorders [17,18]. In addition to the FokI polymorphism, the BsmI polymorphism and the TaqI polymorphism also influence the immune system. Carriers of the BB genotype have higher levels of interferon-gamma (IFN-γ) produced by monocytes than individuals with the Bb or bb genotype. However, the effect of TaqI polymorphism manifests itself in the form of an influence on the level of VDR expression [19,20]. Ethnicity and environmental interactions further modulate VDR function and expression [21].

### 1.4. Hashimoto’s Thyroiditis—Pathogenesis and Antibodies

Hashimoto’s thyroiditis (HT) is an autoimmune chronic disease characterized by self-inflicted thyroid tissue destruction due to an altered adaptive immune system mediated by T cells and lymphocytic infiltration of B and T cells, mostly Th1 CD4+. This leads to fibrosis and thyroid atrophy. With the progression from euthyroid to overt hypothyroidism, previously normal levels of TSH will increase and the free thyroid hormones (FT3 and FT4) levels can decrease. The autoantibodies present are directed against thyroglobulin (anti-Tg) and thyroid peroxidase (anti-TPO). These thyroid autoantibodies levels have a positive correlation with the severity of hypothyroidism. In diagnosis, biochemical determination of the above-mentioned, circulating autoantibody assessment and ultrasonography are used. The autoantibodies most specific and present in 90% of cases are anti-TPO. Due to inflammation, reduced echogenicity in thyroid parenchyma is observed in imaging tests. HT impacts the quality of life. The most prominent is its effect on basal metabolism. Mood disorders, chronic fatigue and body weight changes are common. Symptoms are usually nonspecific, occurring both locally and systematically including systems like the cardiovascular, hematopoietic, reproductive, gastrointestinal and neuropsychiatric system. Pathogenesis is considered in terms of various interactions between genetic and environmental factors but a full etiology is not yet established [2,22].

### 1.5. Vitamin D Deficiency

Vitamin D deficiency is currently one of the leading global health issues. According to the definition, it is characterized by a concentration of calcidiol or 25-hydroxyvitamin D in the blood of <20 ng/mL or <50 nmol/L. An insufficient vitamin D level is considered to be in the range of 20–29.9 ng/mL or 50–75 nmol/L, while its sufficient level is ≥30 ng/mL or ≥75 nmol/L, but it should not exceed 100 ng/mL or 250 nmol/L [23,24,25,26].

A decreased level of vitamin D in serum is closely associated not only with skeletal diseases but also with non-skeletal diseases. These include cardiovascular diseases, cancers, primarily breast, colon, and thyroid cancers, as well as numerous autoimmune diseases: HT, Graves’ disease, systemic lupus erythematosus, Addison’s disease, and rheumatoid arthritis. When the vitamin D level is lower than 10 ng/mL or 25 nmol/L, an increased risk of autoimmune thyroid diseases, particularly HT, is observed [23,24,25].

Vitamin D status and bioavailability depend, among other things, on epigenetic factors. Many studies have demonstrated a link between epigenetic modifications of genes involved in vitamin D metabolism and vitamin D levels. The main epigenetic mechanism is methylation via CpG islands located in the gene’s promoter region, which results in reduced gene expression. In addition to methylation, epigenetic mechanisms also include acetylation and phosphorylation of histone proteins. These mechanisms play a significant role in the variability of vitamin D levels in the population and contribute to the development of vitamin D deficiency [27]. Studies have shown that Europeans currently cope better with low UVB exposure and have adequate vitamin D synthesis. This is due to the higher prevalence of the rs12785878-derived allele, which reduces *DHCR7* gene expression, in the European population compared to African and Asian populations [28]. The *DHCR7* gene is located on chromosome 11 and encodes a reductase involved in the epidermal conversion of 7-dehydrocholesterol to cholesterol. Other single nucleotide variants, such as rs1790349, rs7122671, rs1790329, rs11606033, rs2276360, rs1629220, and rs2282618, may also play a protective role against vitamin D deficiency [27]. The presence of positive evolutionary selection makes Europeans more sensitive to vitamin D than Asian and African populations. This demonstrates that vitamin D status is a trait dependent on environmental and epigenetic factors [28].

### 1.6. Vitamin D Receptor

The VDR is an endocrine intracellular nuclear receptor and a significant signaling molecule that enables the biological effects of the active form of vitamin D [29,30]. The gene encoding VDR is located on chromosome 12 at locus 12q13.11 [1]. On the surface of target organ cells, 1,25(OH)2D binds to VDR, leading to its activation. VDR binds to the retinoid X receptor, forming a heterodimeric complex. This complex binds to the promoter regions of target genes in DNA. This means that the transcriptional activity of these genes is fully regulated and may result in their activation or suppression [23,25,29,31]. For the proper expression of target genes, both VDR activity and the correct concentration of vitamin D in the blood are necessary. If at least one of the components of this mechanism fails, the expression of genes that are necessary, among others, for calcium reabsorption in renal tubules will be impaired [24].

In addition to the genomic mechanism of action, the VDR and retinoid X receptor complex also act through non-genomic pathways. An example is in the immune system, where vitamin D may regulate the binding of VDR to target proteins, including IKK-β (an inhibitor of nuclear factor kappa-B kinase subunit beta) and STAT1 (signal transducer and activator of transcription 1). As a result, vitamin D plays a significant role in the cross-modulation of gene expression involving non-vitamin D ligands, such as tumor necrosis factor-alpha (TNF-α) and IFN-γ [10].

*VDR* gene expression is present in more than half of human tissues. VDR and the active form of vitamin D play a pleiotropic role in the regulation of metabolic pathways, especially the energy metabolism pathway [30]. VDR is also expressed on the surface of cells in the immune system, including dendritic cells, antigen-presenting cells (APCs), and B and T lymphocytes. This allows the receptor to control the proliferation and differentiation of such cells [24,29]. In addition to VDR expression, most cells of the immune system also show expression of the enzyme 1α-hydroxylase (CYP27B1), which enables the conversion of vitamin D precursors into 1,25(OH)2D [2,32].

The complicated organization of the *VDR* gene makes the identification of functional polymorphisms difficult [33]. However, depending on the type of *VDR* gene polymorphism, various functional implications have been described, which may concern the structure and stability of the VDR protein, translation efficiency, or mRNA splicing [21,34]. FokI and TaqI polymorphisms may modify the structure of the VDR protein and the efficiency of mRNA transcription by affecting mRNA splicing. ApaI and BsmI polymorphisms, on the other hand, may reduce *VDR* gene expression and mRNA stability [34,35]. In recent years, studies that analyzed various features of the biological response have been conducted. These studies have identified four levels of organization that allow for characterizing the relationship between VDR polymorphisms and diseases and demonstrating the functionality of VDR polymorphisms [33]:

1. Cellular level—concerns the impact of VDR polymorphisms on transcriptional activity and cell growth.
2. Protein level—concerns the impact of VDR polymorphisms on VDR protein stability, isoforms, protein–protein interactions, and the influence on the regulation of VDR protein levels.
3. Human level—demonstrates the association between VDR polymorphisms and parameters that can be examined using blood serum and calcium homeostasis.
4. mRNA level—concerns the impact of VDR polymorphisms on mRNA stability, isoforms, splicing, and the influence on the regulation of mRNA levels [33].

### 1.7. The Role of Vitamin D in Immune System Regulation

Vitamin D significantly influences the activity of the immune system. It enhances the innate immune response while inhibiting acquired immunity, thus contributing to maintaining immunological tolerance. Innate immunity is enhanced by stimulating the production of pattern recognition receptors, defensin β2, antimicrobial peptide (CAMP), and cytokines in cells [19,36]. 1,25(OH)2D is responsible for the removal of pathogens by inducing chemotaxis and phagocytosis of innate immune cell components. According to recent studies, vitamin D reduces the chance of developing infections by minimizing the propagation of pathogens through the formation of neutrophil extracellular traps [19]. Vitamin D interacts with monocytes, APCs, dendritic cells, and B and T lymphocytes These cells express VDR and 1α-hydroxylase. 1,25(OH)2D affects APCs by inhibiting the surface expression of major histocompatibility complex class II (HLA class II) antigens and molecules involved in co-stimulation. When 1,25(OH)2D interacts with dendritic cells, expression of the costimulatory molecules CD40, CD80, and CD86, as well as human leukocyte antigen (HLA) class II, is reduced [29]. Vitamin D also plays a key role in macrophage maturation. It may also contribute to the transformation of monocytes into macrophages, while its deficiency significantly reduces the antimicrobial activity of monocytes. The effect of vitamin D on monocytes also affects Toll-like receptors 2 and 4 (TLR2 and TLR4), which are located on the surface of monocytes. This inhibits the expression of TLR2 and TLR4 and prevents their excessive activation. The effect of the first action is reduced synthesis of pro-inflammatory cytokines, while the effect of the second action can be observed in the form of inhibition of inflammation [29]. Through paracrine mechanisms, 1,25(OH)2D stimulates macrophages and through autocrine mechanisms it may cause an increase in cathelicidin expression, having antibacterial and antiviral properties [27,37]. Cathelicidin synthesis is stimulated by TLR activation in monocytes and macrophages, which also enhances the expression of VDR and 1α-hydroxylase [36]. It has been suggested that vitamin D prevents an exaggerated response from activated T lymphocytes, which may lead to the development of various autoimmune diseases [30]. Research shows that patients with autoimmune disorders often have vitamin D insufficiencies [8,25,26]. As mentioned before, the Cdx2 promoter of *VDR* gene polymorphism changes overall activity and VDR transcription and through this mechanisms influences active vitamin D activity [27]. In its homozygous form, Cdx2 polymorphism is associated with the diagnosis of asthma [38]. The cells affected by vitamin D in this example are CD4+ T cells. Another example of an autoimmune disease linked to vitamin D is inflammatory bowel disease where the cells affected by this vitamin include B cells and T cells but also dendritic cells and macrophages [36].

## 2. Methods

### 2.1. Protocol

Although a formal review protocol was not pre-published for this study, the methodological approach was guided by the Preferred Reporting Items for Systematic Reviews and Meta-Analysis Protocols (PRISMA-P) and the Joanna Briggs Institute’s scoping review guidelines. The reviewer revised and tailored the protocol components to fit the review’s objectives, basing the report items on the PRISMA extension for Scoping Reviews (PRISMA-ScR).

### 2.2. Data Sources

A thorough electronic search was conducted by the reviewer across multiple databases to find evidence on the subject: PubMed (Medline) and Google Scholar. The search did not extend to additional sites for unpublished sources or gray literature, focusing instead on peer-reviewed, methodologically robust evidence. The search strategy employed various combinations of MeSH terms and Boolean operators, combining multiple formats and synonyms of main concepts: “vitamin D”, “Hashimoto”, “vitamin D receptor (VDR)”, “polymorphism” and “supplementation”. The search strategy was slightly adjusted to accommodate the features of each database or optimized to yield the broadest range of results while maintaining relevance to the topic. Each concept was represented by multiple synonyms and abbreviations, using Boolean operators OR and AND to ensure comprehensive retrieval of relevant sources from the targeted databases. The search strategy was slightly adjusted to fit the specific features of each database and to maximize relevant results. For example, the search string used in the PubMed database was as follows: vitamin D AND (vitamin D receptor OR VDR) AND Hashimoto AND polymorphism AND supplementation. The search covered publications between from March 2018 to November 2024.

### 2.3. Eligibility Criteria

As a scoping review, the study employed inclusive eligibility criteria to identify the maximum number of relevant sources showing a relationship between the vitamin D receptor and its polymorphisms and various diseases, including autoimmune diseases such as HT, the presence of specific polymorphisms and the severity of HT as well as the response to vitamin D supplementation and articles discussing the role of vitamin D in the immunopathogenesis of HT. Papers published in languages other than English were excluded to ensure review accuracy and avoid potential translation errors. Additionally, case reports, book chapters, and conference papers were excluded to ensure the inclusion of only high-level primary evidence sources.

### 2.4. Source Selection

The reviewers screened the retrieved sources in two phases. In the first phase, duplicates were removed using free online software and then manually double-checked. After duplicate removal, the titles, abstracts, and keywords of the sources were screened to identify those mentioning the two main concepts and to exclude non-English sources. In the second phase, the abstracts of the remaining sources were reviewed to determine their relevance to the review topic and to identify whether it covers with the main concept of review. This screening helped the reviewers select articles with methodologies aligned with the review’s aims and inclusion criteria.

**Figure 1:**
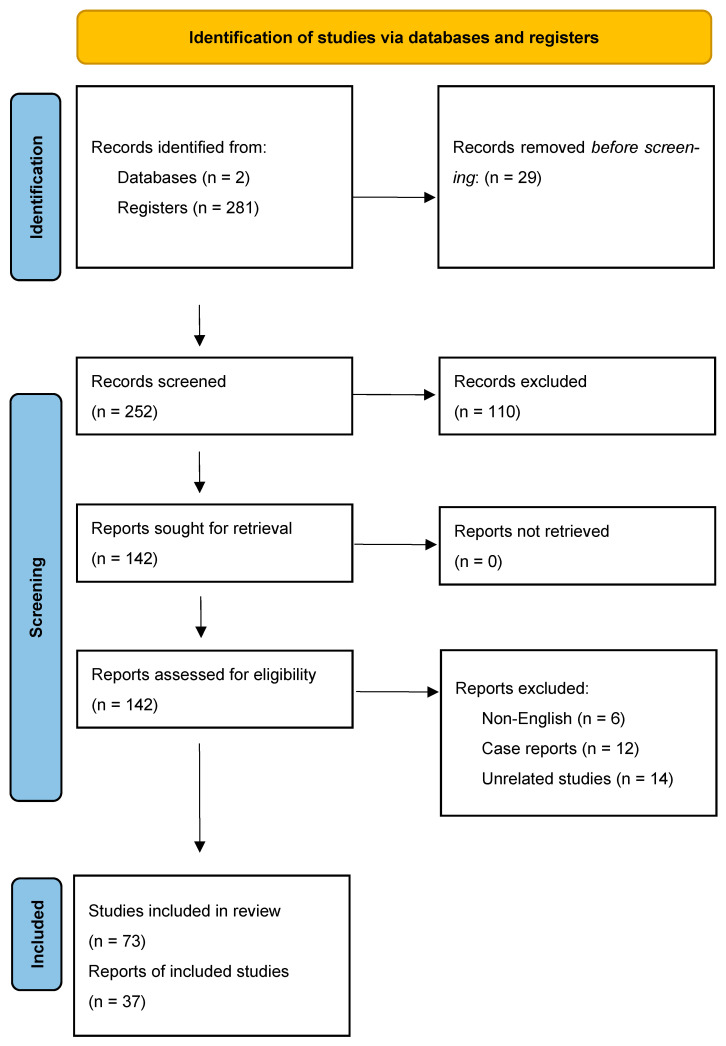
PRISMA flow diagram. Prisma note: Prospero International Prospective Register of Systematic Reviews [39]. Available online: https://www.crd.york.ac.uk/prospero/ (accessed on 19 October 2025).

The initial database search yielded 281 results, which were filtered in the first phase by removing duplicates. In the next phase, 252 sources underwent full-text analysis for relevance and methodological rigor, leading to the exclusion of 110 sources. Reports assessing for eligibility excluded non-English articles, case reports and unrelated studies. Screening resulted in a total of 110 articles included in the scoping review (Figure 1).

## 3. Genetic Causes of Different Vitamin D Receptor Densities—Gene Polymorphisms and Clinical Implications

The aim of this section is to present: (1) factors that may influence VDR expression, (2) the diversity of studies, populations, and methodologies assessing the relationship between *VDR* gene polymorphisms and vitamin D levels in the context of HT and other various diseases, and (3) to demonstrate the association between specific types of polymorphisms and the severity of HT and the response to vitamin D supplementation.

The density of VDR may be influenced by several factors related to genes. The most commonly studied factor is *VDR* gene polymorphisms, especially with regard to various diseases. The vitamin D status has been linked to chronic diseases including autoimmune diseases but also osteoporosis and cancers. Polymorphisms may affect the translation start site of VDR protein or affect the gene’s mRNA stability and even alter miRNA binding. The less translated and transcribed VDR gene is, the less VDR protein there is, which affects the receptor density. Genome-wide studies additionally show other factors having such an influence. Those factors that could affect VDR densities include gene expression regulation such as higher or lower promoter activity modulated by regulatory genes that code, for example, transcription factors or signaling molecules. There are polymorphisms of genes coding the transport proteins. Additionally there are coactivation proteins or molecules that have an impact on production or activation of vitamin D or mechanisms secondarily affecting its expression. DNA methylation or histone modifications are also of importance [27].

This review focuses on VDR polymorphisms. Polymorphisms of vitamin D receptors (VDRs) are identified as genetic factors influencing on vitamin D status of an individual. The *VDR* gene contains a total of six promoter regions and consists of 8 exons, which are important when it comes to *VDR* gene polymorphism. Polymorphic sites of VDR are named after restriction endonuclease enzymes recognizing them. The four most well-characterized are FokI (rs10735810 also known as rs2228570), TaqI (rs731236), ApaI (rs7975232) and BsmI (rs1544410) [27].

FokI influences the structure of VDR resulting in the production of a shortened protein but is characterized by an increased transcriptional activity. There is also an association between lower levels of 25(OH)D levels in serum and FokI. Regardless of whether polymorphisms lead to decrease in VDR density, a decrease in VDR functionality will also affect the calcitriol’s action. TaqI is a polymorphism affecting the *VDR* gene methylation status. Methylation is a form of silencing, interfering with gene transcription. BsmI affects transcript stability. This means the next step will be affected and translation to protein status will be different. This will result in different VDR density status. VDR promoter region polymorphisms are less well-known. GATA (rs4516035) and Cdx2 (rs11568820) are polymorphisms located, respectively, downstream and upstream of exon 1 and are responsible for a decreased activity of the promoter [27] (Table 1).

Table 1 shows *VDR* polymorphisms and their effects and results regarding the VDR. Polymorphisms GATA and Cdx2 are often evaluated together.

After discussing the molecular mechanisms, the clinical context should be analyzed. It should be noted that numerous studies have shown the impact of VDR polymorphisms on HT (Table 2) and on diseases other than HT (Table S1 in Supplementary Materials). Population and methodological differences are important factors that may influence the interpretation of results. Different research strategies and statistical limitations, despite the number of conducted investigations, may contribute to inconsistencies in the final outcomes.

A study conducted by Marini et al. suggests that the FokI CC genotype (shorter VDR protein) does not lead to greater NF-κB transcriptional activity in immune cells. There are no differences in FokI genotype distribution between juvenile idiopathic arthritis patients and healthy controls, or between disease activity groups. This supports the hypothesis that low vitamin D levels and other genetic factors including the FokI CC genotype can be a trigger in the development of autoimmune diseases [40].

**Table 2:** Comparative table of the characteristics of individual studies on the impact of VDR polymorphisms on the development and severity of HT and their correlation with serum 25(OH)D levels.

Author and Year of Investigation	Population Backgrounds	Sample Size by Case Group	The Disease Under Investigation	Type of Investigation	Results and Outcomes
Kamyshna et al., 2021 [1]	West-Ukrainian	153 participants divided into three groups: - 1st group—16 participants (mean, 47.30 ± 12.27 years), - 2nd group—65 participants (mean, 46.72 ± 15.49 years), - 3rd group—72 participants (mean, 45.02 ± 13.65 years). No data regarding gender distribution.	- 1st group—postoperative hypothyroidism, - 2nd group—hypothyroidism caused by Hashimoto’s thyroiditis, - 3rd group—Hashimoto’s thyroiditis with elevated levels of anti-TPO and anti-Tg antibodies	cross-sectional study	1. The rs2228570 polymorphism of the *VDR* gene is associated with a significant decrease in 25(OH)D levels in patients with postoperative hypothyroidism and HT compared to the control group. 2. No significant differences were found in the frequency of the FokI polymorphism between the three patient groups studied. 3. In each of the three patient groups, the 25(OH)D level was significantly lower, regardless of genotype (AA, AG, and GG), compared to the control group. The odds ratio (OR) test shows no differences in the distribution of the AA, AG, and GG genotypes between the study group and the control group. The AG genotype was the most frequent, followed by the AA genotype, while the GG genotype was the least common (*p* > 0.05). There were no differences in the frequencies of the A allele (*p* > 0.05) and the G allele (*p* > 0.05) between the groups.
Hanna et al., 2021 [41]	Egyptian	112 participants, including 107 females and 5 males, aged over 18 years (31.5–51 years)	Hashimoto’s thyroiditis	cross-sectional study	1. The AA FokI genotype has a significantly higher probability of being associated with HT. In the control group, this genotype was not present at all. 2. The average level of 25(OH)D in HT patients with the AA FokI genotype was higher than in patients with the AG or GG FokI genotypes (*p* = 0.037).
Maciejewski et al., 2019 [42]	Caucasian Polish	223 participants, including 213 females and 10 males (mean, 46.88 ± 13.50 years)	Hashimoto’s thyroiditis	case–control study	1. No significant association was found between the VDR polymorphisms rs2228570, rs1544410, rs7975232, and rs731236 and the risk of HT. In both groups, the genotype distributions of the studied VDR polymorphisms were in accordance with the Hardy–Weinberg equilibrium (*p* > 0.05). 2. The distributions of alleles and genotypes of the studied *VDR* polymorphisms did not differ significantly between patients with HT and the control group. 3. A weak association was observed between the rs1544410 (*p* = 0.03) and rs7975232 (*p* = 0.04) polymorphisms and thyroid volume in patients with HT—value of D prime between the loci: 0.95 (0.89–0.98).
Mestiri et al., 2020 [43]	Tunisian	162 participants divided into two groups: - 1st group—106 participants, - 2nd group—56 participants, including both males and females	- 1st group—Hashimoto’s thyroiditis, - 2nd group—Graves’ disease	case–control study	1. It has been shown that the rs7975232 polymorphism is not associated with susceptibility to HT and Graves’ disease. The AA genotype was observed more frequently in patients with HT and Graves’ disease under 40 years of age compared to those over 40 years of age with *p*-values of *p* = 0.02 and *p* = 0.03, respectively. 2. The rs7975232 polymorphism is not associated with gender, age, or the presence or absence of anti-TPO and anti-Tg antibodies in patients with HT. 3. VDR rs7975232 polymorphism may be a prognostic factor in predicting the severity of HT.
Siddiq et al., 2023 [44]	No data	72 participants, including 56 females (mean, 37 ± 11 years) and 16 males (mean, 33 ± 6 years)	Hashimoto’s thyroiditis	case–control study	1. Patients with HT had significantly lower levels of vitamin D compared to the control group. 2. The rs7975232 polymorphism is significantly associated with disease progression and exacerbation, whereas no such correlation was found for the rs1544410, rs731236, and rs2228570 polymorphisms in the case group.

Investigations on the impact of various VDR polymorphisms on the development of HT yield inconsistent results when comparing ethnic affiliation and population origin of the case group [43,44]. These discrepancies may be due to environmental factors, differences in lifestyle, or different dietary patterns [44].

While study results across different populations and sample sizes are of importance, some meta-analyses and systematic reviews provide more generalized and systematic comparison. Gao and Yu’s meta-analysis included 24 independent studies on the association between the risk of autoimmune thyroid diseases (Graves’ disease and HT) and the VDR polymorphisms rs731236 (TaqI), rs1544410 (BsmI), rs2228570 (FokI) and rs7975232 (ApaI). The researchers used crude odds ratios and confidence intervals to assess the strength of association of allele polymorphisms and the risk. The main populations analyzed were Asian, European and African. Ethnicity greatly influenced the associations for different VDR polymorphisms. The rs731236 (TaqI) polymorphism was associated with a reduced risk of autoimmune thyroid diseases in Asian and African populations but not in Europeans. Polymorphism BsmI, however, showed a statistically significant association with reduced risk in Europeans (in homozygote comparison and dominant models) and Africans (in homozygote comparison and recessive models) but also an increased risk of autoimmune thyroid diseases in Asians (in heterozygote and dominant models). FokI showed decreased risk in Asians but no significant connection to Europeans or Africans. ApaI showed increased risk in Africans but no significant connection to Europeans or Asians. In terms of HT risk only, in the general population, rs731236 (TaqI) (7 studies, 895 cases of HT and 861 controls) and rs2228570 (FokI) (7 studies, 839 HT cases and 788 controls) showed a statistically significant protective effect reducing the risk of HT (*p* = 0.005; *p* = 0.03, respectively). BsmI (8 studies, 1009 HT cases and 1089 controls) and ApaI (6 studies, 757 HT cases and 812 controls) showed no statistically significant association with HT risk [45].

Moreover, according to a 2022 systematic review and meta-analysis by Usategui-Martín et al. that aimed to assess whether single nucleotide polymorphisms (SNPs) in the *VDR* gene modify the body’s response to vitamin D supplementation, TaqI (genotype Tt+tt) and FokI (genotype FF) genetic variants have a significant impact (*p* = 0.02; *p* < 0.001, respectively) on the better effect on vitamin D supplementation, while BsmI and ApaI showed no significant association. The mechanisms responsible for this impact are as follows. TaqI is associated with increased VDR mRNA stability. The FokI polymorphism affects VDR protein translation and its F allele is associated with translation of the more active VDR protein [46].

Polymorphisms of the *VDR* gene are promising for clinical relevance. Response to vitamin D supplementation may vary significantly between individuals, and this is due to different genetic variants in the *VDR* gene. *VDR* polymorphisms may alter VDR activity. The association between specific types of *VDR* polymorphisms and response to vitamin D supplementation has been the subject of numerous studies, but studies focusing on genetic association analysis have yielded conflicting results, and this relationship remains unclear [19,46]. Although there are many studies showing a link between specific types of *VDR* polymorphisms and the response to vitamin D supplementation, there are currently no studies that directly assess the ethical implications of genetic testing in this context. There are, however, findings that address the risks associated with the introduction of genetic testing when it does not provide significant clinical benefits, but these studies primarily focus on nutrigenetics and personalized nutrition. In this area, the availability of tests, the interpretation of their results, and the insufficient training of staff may pose challenges [47].

With technological advances and increased access to personal data, encoding data and addressing privacy threats has become more difficult. The use of Big Data approaches to data collection and analysis, as well as the trend toward increased data sharing, have also created challenges related to providing patients and research organizations with appropriate information about how their data may be shared and used, as well as maintaining privacy protection. Furthermore, polymorphisms are heritable, so genetic results have implications not only for the individual being tested but potentially for family members or groups with genetic ties to that individual. Therefore, efforts to address ethical issues related to privacy or informed consent may require consideration of interests beyond the individual. Moreover, the types of issues that would need to be addressed in the informed consent process in a way that is understandable to the patient include penetration, certainty, and false positive rates. Such aspects are of great importance to patient’s correct understanding of the results, which then lead the patient to changes in personalized nutrition and proper supplementation. The value of research results focusing on polymorphisms is influenced by their level of diagnostic standardization, which, for some polymorphisms, may not yield results with the expected clinical significance. It is also worth noting that patients may consent to access their medical records by external healthcare providers, or even employers, without realizing that their clinical genetic data may be contained in these records and therefore accessible to third parties, potentially leading to abuse of these records [48].

In addition to HT, genetic variants of the *VDR* have also been shown to be associated with numerous diseases, including: susceptibility to type 2 diabetes with coronary artery disease [49], with the risk of several cancers, including colon cancer. However, these associations differ across populations, and their results are ambiguous [50,51]. The findings of Divanoglou et al.’s research conducted on 98 subjects of a Greek homogeneous rural population show the cumulative effect of specific polymorphisms of the VDR genes that may regulate vitamin D levels, explaining partly a vitamin D deficiency paradox in sunny regions [52]. In a single cohort study, Apaydin et al. showed that VDR gene polymorphisms are independently associated with COVID-19 severity and patient survival [53].

It should be noted that the association between *VDR* polymorphisms and autoimmune diseases definitely differs across ethnic populations. The impact of vitamin D receptor polymorphisms on other diseases has also been described in several reviews [54,55,56].

### 3.1. Overview of Study Results by Population Ethnicity and Sample Size

The results of the studies presented in Table 2 and Table S1 [40,49,50,51,57,58,59,60,61,62,63,64,65,66,67,68,69,70,71] (in Supplementary Materials) demonstrate significant discrepancies in the association of VDR polymorphisms with disease susceptibility, including HT and vitamin D status, as well as in the relationship between vitamin D deficiency and disease susceptibility.

Significant associations in these areas were observed in populations with moderate, and small sample sizes. Studies in Table 2 that showed an association include: Kamyshna et al. [1] (153 participants), Hanna et al. [41] (112 participants), and Siddiq et al. [44] (72 participants). Participants in these studies were from Egypt and from Ukraine.

No significant associations were observed in both moderate and small sample sizes. This applies to the studies from Table 2: Maciejewski et al. [42] (223 participants) and Mestiri et al. [43] (162 participants). The population background of participants were Tunisian and Caucasian Polish.

Comparative analysis of studies reporting and not reporting significant associations suggests that the discrepancies observed in Table 2 and Table S1 (in Supplementary Materials) are not directly related to the sample size, as significant associations or their absence were observed in both small and large groups. Factors that are more likely to influence the occurrence of discrepancies include ethnicity, differences in the selection of polymorphisms analyzed, and the specificity of the diseases analyzed, including HT. However, the number of studies is again too small to draw objective conclusions. Furthermore, methodological heterogeneity may further complicate comparative analysis. The studies presented in both tables included case–control and cross-sectional studies. Moreover, some studies analyzed single polymorphisms, whereas others analyzed several polymorphisms simultaneously, which could have contributed to the divergent results.

The associations between the different types of polymorphisms studied also vary depending on the ethnicity of the studied population.

Significant associations between the FokI polymorphism (rs2228570) and susceptibility to HT were observed in Ukrainian (Kamyshna et al. [1]) and Egyptian (Hanna et al. [41]) populations. In contrast, the study conducted by Maciejewski et al. [42] in a Caucasian (Polish) population did not demonstrate such an association. These findings suggest that the FokI polymorphism may be linked to a higher risk of the disease in African and Eastern European populations than in Caucasians.

Regarding the ApaI polymorphism (rs7975232), Siddiq et al. [44] reported an association between HT and disease progression, although the ethnic background of the study population was not specified. In the study conducted by Mestiri et al. [43] on the Tunisian population, ApaI polymorphism was not associated with an increased risk of HT, but it may serve as a prognostic factor for the disease severity. However, in the study by Maciejewski et al. [42], similarly to the FokI polymorphism, no association was observed in the Caucasian population. Based on these findings, it can be inferred that the ApaI polymorphism may play a more significant role in the progression and severity of the disease in the African population than in the Caucasian population.

Studies of the BmsI (rs1544410) and TaqI (rs731236) polymorphisms were conducted only by Maciejewski et al. [42] in a Caucasian population and showed no association between with susceptibility to HT. As populations from other continents were not included in studies of the BsmI and TaqI polymorphisms, their association with disease susceptibility in other populations remains unconfirmed.

These findings underscore the need for further investigations including participants from more ethnically diverse populations and varying sample sizes. Ideally, studies conducted within specific ethnic groups, with small, moderate, and large sample sizes, would allow for a meaningful comparative analysis of study results.

### 3.2. Overview of Study Results by Evidence Strength and Heterogeneity

The strength of evidence regarding the association between different *VDR* polymorphisms and susceptibility to HT varies across studies, being statistically significant (*p* < 0.05) or non-significant (*p* > 0.05), depending on the investigation. In the studies conducted by Mestiri et al. [43] and Hanna et al. [41], based on the reported *p*-values, this association can be considered statistically significant. In contrast, the *p*-values reported by Maciejewski et al. [42] and Kamyshna et al. [1] indicate no significant association between the presence of certain *VDR* polymorphisms and susceptibility to HT or disease severity. The odds ratio values were not reported in all studies, which limits the possibility of a comprehensive analysis of the effect size.

The heterogeneity of the results presented in Table 2 is high, which can be attributed to differences in the analyzed *VDR* polymorphisms, ethnic backgrounds of the studied populations, discrepancies in the type of study design, and differences in the sample sizes across studies.

Based on the analysis of the evidence strength and heterogeneity, it can be concluded that the observed associations are largely specific to a specific population or individual polymorphism, which prevents the generalization of conclusions to the overall population.

## 4. Vitamin D and Immune Reactions in Hashimoto’s Thyroiditis

**Figure 2:**
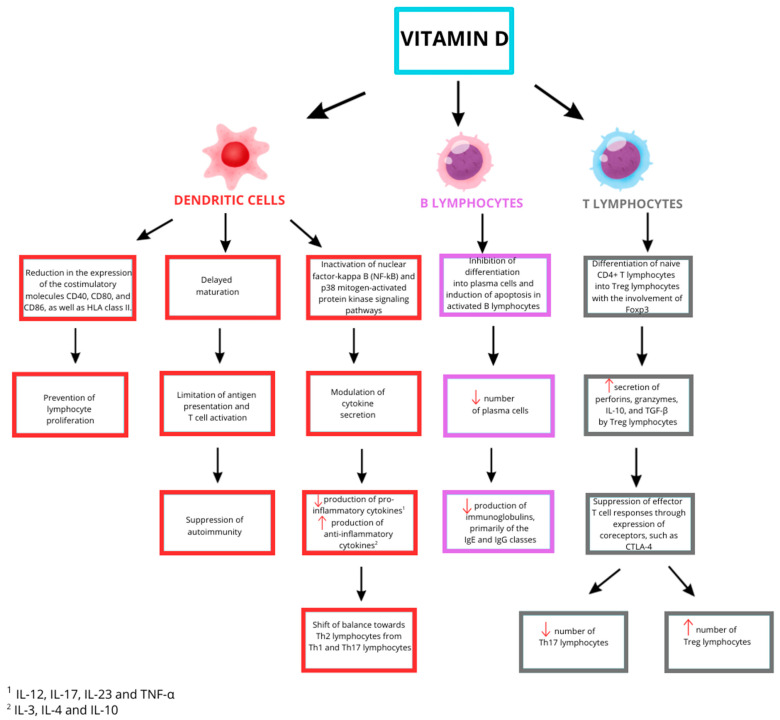
The role of vitamin D in the immunological processes occurring in Hashimoto’s thyroiditis [2,23,29,32,72,73]. The down arrow indicates a decrease and the up arrow indicates an increase.

Vitamin D inhibits many components of the immune response in Hashimoto’s thyroiditis, thus exerting an immunosuppressive function. The effect of vitamin D on immune system cells such as dendritic cells, B lymphocytes and T lymphocytes is presented in Figure 2. Vitamin D prevents the maturation of dendritic cells, leading to their lack of activation. This impairs antigen presentation and T lymphocyte activation, which typically involves dendritic cells.

This is a significant aspect contributing to the suppression of autoimmunity. The influence of vitamin D on dendritic cells is also manifested in the modulation of cytokine secretion. There is a decrease in the release of pro-inflammatory cytokines such as interleukin (IL): IL-12, IL-17, IL-23, or TNF-α, and an increase in the secretion of anti-inflammatory cytokines, including IL-3, IL-4, and IL-10. This contributes to a shift in the phenotype and balance towards Th2 lymphocytes away from Th1 and Th17 lymphocytes [29,72].

Vitamin D is also responsible for the differentiation of naïve CD4+ T cells into regulatory T cells (Treg), stimulates their proliferation, and increases the secretion of IL-10, transforming growth factor-beta (TGF-β), granzymes, and perforins produced by Treg lymphocytes [29,32]. Foxp3 plays an important role in the development of Treg lymphocytes [73]. Thanks to the secreted substances and the expression of co-receptors, such as CTLA-4, which prevent antigen presentation, Treg cells are able to suppress effector T cell responses [32]. As a consequence of the processes described above, there is a reduction in the number of Th17 cells and an increase in the number of Treg cells. Restoring the balance between these cell populations is one of the goals that may contribute to improving the treatment of autoimmune diseases. In cases of vitamin D deficiency, increased secretion of TNF-α stimulates thyroid cells to release the CXCL10 cytokine. The result is positive feedback and the initiation of an autoimmune process that becomes permanent over time. Vitamin D also inhibits NF-κB and p38 MAPK signaling in dendritic cells, further reducing pro-inflammatory cytokine secretion [23,29].

In B lymphocytes, vitamin D limits proliferation and differentiation into plasma cells, and memory B cell production, while inducing apoptosis in activated B lymphocytes. The decreased number of plasma cells results in lower immunoglobulin production, mainly of the IgE and IgG classes. In the case of vitamin D deficiency, this process is not inhibited, leading to high immunoglobulin levels, ultimately causing thyroid cell damage and the development of HT. Autoantibodies produced by B lymphocytes play a dominant role in the pathophysiology of autoimmune diseases. Therefore, the above-described mechanism for controlling the proliferation and activation of B lymphocytes may have a significant impact on autoimmune diseases [2,29,32].

Regulatory B lymphocytes, which are involved in immune tolerance through the production of cytokines such as IL-10, IL-35, and TGF-β, are also modulated by vitamin D [72].

Nodehi et al. conducted a double-blind, placebo-controlled study involving 48 female patients with HT, divided into two groups of 24 participants each. Both groups received weekly oral treatment with 50,000 international unit (IU) of cholecalciferol or placebo for 3 months. The study results showed that vitamin D supplementation positively affects the immune system in patients with HT by reducing the Th17/Treg ratio. No significant differences were observed in the numbers of Th1, Th2, Treg, and Th17 cells between the vitamin D and placebo groups, although an increase in IL-10 expression was observed in both groups [29,74]. In the study by Botelho et al. [75], 88 participants with HT and 71 euthyroid healthy controls were assessed. Participants were evaluated for markers of thyroid autoimmunity and serum vitamin D levels. The study demonstrated a relationship between the levels of cytokines TNF-α, IL-5, and IL-17 and vitamin D levels, confirming an association between vitamin D and autoimmunity in HT [29].

Although the results of these studies provide information on the immunomodulatory effects of vitamin D, the small sample sizes and the short study duration limit the generalizability of the results.

Further investigations should clarify the optimal vitamin D levels required to prevent the development of autoimmune diseases, as such levels have not been clearly defined despite numerous studies [72]. Standardization of the vitamin D supplementation protocols and methods for measuring serum vitamin D concentration is essential [32]. Further investigations should also assess the influence of environmental factors, such as exposure to UV radiation and intestinal microbiota, on the immunomodulatory effects of vitamin D. A review of the current literature reveals a lack of clear evidence regarding the effects of vitamin D supplementation across different populations, as available studies generally include small and heterogeneous participant groups.

## 5. Relationship Between Vitamin D Level and Frequency of Thyroid Diseases and Antibody Titer

The effect of vitamin D on the functioning of the thyroid gland is still not fully understood. However, numerous studies have shown that genetic disorders involving the polymorphism of genes related to vitamin D metabolism and signaling correlate with an increased risk of autoimmune thyroid diseases [76].

The most important correlation was found between vitamin D deficiency and hypothyroidism. When comparing the polymorphism of 25(OH)D-related genes in patients with Hashimoto’s thyroiditis, Graves’ disease, and autoimmune hyperthyroidism, vitamin D has the greatest influence on the development of Hashimoto’s thyroiditis [77]. TSH is negatively correlated with 25(OH)D levels, and FT3 and FT4 levels are positively correlated with 25(OH)D levels [78]. An overview of the selected studies examining the supplementation of vitamin D in thyroid diseases and the relationship between vitamin D status and thyroid function is presented in Table 3.

A study by Kishore Kumar Behera conducted in 2020 among 23 patients with Hashimoto’s thyroiditis in Bhubaneswar, India, showed vitamin D3 deficiency in 22 of them. This confirms the relationship between a deficiency of this vitamin and hypothyroidism. The subjects were advised to supplement with 60,000 IU of vitamin D3 once a week for 8 weeks and once a month for the next 4 months. After the normalization of the vitamin level, most of them showed a decrease in TSH levels, as well as an increase in the level of anti-TPO [79].

A similar study was conducted in 2021 at the Collegium Medicum in Bydgoszcz by Marcin Gierach and Roman Junik in a group of 370 people, which showed that in the group of women, low levels of vitamin D in the blood are associated with high TSH levels. Gierach et al. compared 125 healthy patients, 111 with hypothyroidism and 134 with Hashimoto’s thyroiditis. Additionally, a negative correlation was found between vitamin D and the level of anti-TPO and anti-Tg antibodies in each of the studied groups of women [80].

It has been shown that a significant reduction occurs in the levels of anti-Tg and TSH in the group of patients who were given vitamin D compared to the beginning. This confirms that vitamin D supplementation may be helpful in ameliorating disease activity in patients with HT. However, further well-controlled and longitudinal studies are needed to determine whether it may be introduced into clinical practice [72].

There is a suggestion that vitamin D reduces autoantibody titers in patients with HT [81]. The active form of the vitamin D significantly reduces anti-TPO titer, while treatment lasting more than 12 weeks results in more effective reduction in anti-TPO levels and more significant increase in FT4 and FT3 levels in patients with HT. Vitamin D supplementation may have beneficial effects on patients with HT by modulating the immune response and improving thyroid function [82]. Not only does supplementation improve the patient’s prognosis, but a well-balanced diet may also help prevent vitamin D deficiency and improve the quality of life of patients with HT, especially those who are in the later stages of the disease, characterized by greater metabolic imbalance [83]. In turn, the Cvek study indicates that there is no association between vitamin D and HT; however, there may be a subtle decrease in vitamin D levels associated with overt hypothyroidism [84].

In the case of healthy patients, vitamin D deficiency may be associated with impaired sensitivity to thyroid hormones, so monitoring the level of this vitamin should be recommended in high-risk patient groups. It has been shown that there is a relationship between 25(OH)D levels and sensitivity to thyroid hormones in euthyroid patients [85].

In turn, in a retrospective observational cohort study conducted in Seoul, the impact of vitamin D supplementation on the prognosis of differentiated (papillary or follicular) thyroid cancer was demonstrated. They showed that ensuring proper vitamin D concentration may be a beneficial factor ensuring improved survival in patients with these cancers [86].

The importance of vitamin D in the context of the proper functioning of the thyroid gland and hormonal balance is not fully known. Numerous research studies have proven the existence of a correlation between the concentration of vitamin 25(OH)D and thyroid hormones [72,77,78,82,85,87]. This is due to the presence of similar steroid receptors for both of these chemical compounds. Many studies have shown that vitamin D supplementation and constant monitoring of its level may be a beneficial prognostic factor protecting against the development of autoimmune diseases, in particular, Hashimoto’s thyroiditis [82,85,87].

**Table 3:** Comparative table of the characteristics of individual studies on vitamin D supplementation in thyroid diseases and the relationship between vitamin D levels and antibodies titer.

Author and Year of Investigation	Population Backgrounds	Sample Size by Case Group	The Disease Under Investigation	Type of Investigation	Results and Outcomes
Behera K.K., 2020 [79]	Bhubaneswar, India	23 patients with HT (22 with vitamin D3 deficiency)	Hashimoto’s thyroiditis	intervention, prospective	Vitamin D3 supplementation (60,000 IU/week for 8 weeks, then 1×/month for 4 months) decreased TSH and increased vitamin D levels; confirmed link between deficiency and hypothyroidism.
Gierach M., Junik R., 2021 [80]	Collegium Medicum, Bydgoszcz	370 people (125 healthy, 111 hypothyroid, 134 with Hashimoto); mainly women	Hypothyroidism and Hashimoto’s thyroiditis	observational, cross-sectional	Low vitamin D levels correlated with high TSH and elevated anti-TPO and anti-Tg antibodies.
Chahardoli R., 2016 [72]	Erfan Hospital and Imam Khomeini Hospital Complex, Tehran, Iran	42 women with HT	Hashimoto’s thyroiditis	double blind, randomized clinical trial	Vitamin D supplementation decreased TSH and anti-Tg levels.
Zhou L, 2023 [85]	Chao-yang Hospital, Bejing	3143 euthyroid adults (1849 with vitamin D deficiency)	Euthyreosis	observational	Vitamin D levels were associated with tissue sensitivity to thyroid hormones.
Ahn, J, 2023 [86]	Seoul, South Korea	Patients with thyroid cancer (papillary/follicular)	Differentiated thyroid cancer	retrospective, observational (cohort)	Adequate vitamin D levels were associated with better prognosis and survival in patients.

## 6. The Role of Vitamin D Supplementation in Preventing the Development of Autoimmune Diseases

In recent years, many benefits associated with vitamin D supplementation have been noticed. It is recommended not only due to the regulation of calcium-phosphate metabolism responsible for bone mineralization, but also due to its protective effect in the development of autoimmune diseases, as well as cancer and neurodegenerative diseases [88,89].

Vitamin D deficiencies are very common, and improving vitamin D3 levels in order to prevent the development of related diseases should be one of the main goals of health policy [4]. People from risk groups are particularly susceptible to deficiency: obesity, chronic kidney or liver disease, people with impaired absorption of fats from the gastrointestinal tract, e.g., after bariatric surgery, and people undergoing treatment with glucocorticosteroids and anticonvulsants [90].

Vitamin D comes in four main forms: cholecalciferol, ergocalciferol, calcidiol and calcitriol. However, cholecalciferol is the form of vitamin D that should be considered for supplementation purposes, because the other forms are not sufficiently stable and nutritious [91].

Early prevention of vitamin D deficiency in the general population of healthy people involves oral administration of cholecalciferol in an appropriate dose:

- in full-term newborns and infants—400 IU/day [4],
- in prematurely born children—800 IU/day (until they reach the corrected age of 40 weeks) [4],
- in children and adolescents from 1 to 10 years of age—600–1000 IU/day [4],
- in children and adolescents from 11 to 18 years of age—1000–2000 IU/day [4],
- in adults—800–2000 IU/day [4],
- in seniors over 75 years of age—2000–4000 IU/day [4].

These dosages are based on the general population. Among people exposed to the sun without protection against UV radiation for 30–45 minutes between 10 a.m. and 3 p.m. from May to the end of September, supplementation is not necessary [4]. However, it should be remembered that the demand for vitamin D depends not only on age, but primarily on body mass index, ethnic origin and genetic conditions [92]. It is necessary to monitor the effectiveness of therapy.

Vitamin D deficiency may disturb the human immune balance and endothelial stability. It has been shown that the supply of vitamin D has a beneficial effect on the differentiation and maturation of macrophages, dendritic cells and lymphocytes. They contain: VDR and 1α-hydroxylase, i.e., the activating enzyme. VDRs, like enzymes regulating calcium and phosphorus metabolism, are expressed in various types of immune cells. It is still not clear what level of vitamin D may ensure the proper functioning of the immune system. It has also not been demonstrated whether vitamin supplementation protects against the development of autoimmune diseases or whether it has a beneficial therapeutic effect in established disease [75,93,94,95,96,97,98,99,100,101].

It is essential to monitor both the effectiveness of therapy and the concentration of the vitamin D in the body. Patients should not take more than 4000 IU per day. Administration of higher doses may result in hypercalcemia, hypercalciuria and accumulation of deposits in soft tissues, but overt hypercalcemia is rare and is most often associated with endogenous overproduction of metabolites. Most often, vitamin D overdose occurs by an endogenous mechanism. The lowest dose that can cause hypercalcemia in healthy adults is a chronic vitamin D intake of 3800 IU per day. In a study conducted among 373 healthy 62-year-old individuals with excess vitamin D, 400 IU, 4000 IU, and 10,000 IU per day were administered for 3 years. Hypercalcemia was diagnosed in 0%, 3%, and 9% of patients in the groups receiving 400 IU, 4000 IU, and 10,000 IU per day, respectively. Hypercalciuria was more common in all three groups, reaching 17%, 22%, and 31%, respectively. Based on this study, the higher the vitamin D intake, the greater the risk of developing complications. However, after exceeding 4000 IU per day, the risk of both hypercalcemia and hypercalciuria increases significantly. The risk of these complications also increases with long-term vitamin D supplementation [102]. In recent years, the safety of the upper limit of normal of 4000 IU per day has been questioned, and no consensus has been reached regarding a safe upper dose [102,103]. Few studies have evaluated the effects of vitamin D intake of 10,000 IU per day or more over a sufficiently long period, defined as at least 12 months. Studies meeting these criteria reported a low incidence of adverse effects. Because reported cases of vitamin D overdose vary widely, it is impossible to calculate an average dose that will definitely cause toxic effects, and it is also impossible to determine the time after which such adverse effects might occur. A case report by doctors at the University of Modena describes a 56-year-old woman who took an average daily dose of 130,000 IU of vitamin D over a period of 20 months, which resulted in hypercalcemia and acute kidney injury. It took about 6 months for renal function to normalize, and 18 months for serum vitamin D levels to return to the reference range [104]. In contrast, a study by Carla Pizzini from the American University of Beirut Medical Center, Lebanon describes the case of a 22-month-old girl who developed nephrocalcinosis as a consequence of an overdose of vitamin D contained in a dietary supplement purchased online. It initially manifested as polydipsia, polyuria and impaired growth. After treatment, vitamin D levels leveled off within a year and growth normalized, but nephrocalcinosis was not cured [105]. These data indicate that the results of studies on vitamin D supplementation and its potential adverse effects are inconsistent, as the risk of toxicity depends not only on the dose but also on the duration of administration, treatment regimen, individual characteristics of such as age and sex, and the source of vitamin D, making it difficult to establish clear, universal guidelines for safe supplementation. Vitamin D is essential for bone health and immune regulation, and its deficiency is widespread, especially in high-risk groups. Cholecalciferol (vitamin D3) is the preferred form for supplementation, with doses adjusted by age and individual needs. While supplementation within recommended limits is safe, excessive intake above 4000 IU per day increases the risk of hypercalcemia and other complications. Regular monitoring of vitamin D levels is crucial to ensure both safety and effectiveness of therapy [102,103].

Clinical implications of molecular mechanisms of VDR polymorphisms on autoimmune disease prevention and vitamin D supplementation involve identifying individuals genetically predisposed to a poor response to vitamin D, which would require individual adjustment of the supplement dosage in order to achieve the tolerogenic and protective effect of vitamin D and prevent the development of autoimmune diseases. While vitamin D supplementation generally exerts immune-supporting and anti-inflammatory effects, specific *VDR* genetic variants can alter the strength and efficacy of this response. Some polymorphisms lead to higher serum vitamin D levels after supplementation, whereas others are associated with altered immune responses or susceptibility to autoimmune diseases [36,88,106,107,108].

A meta-analysis of 23 studies (Bagheri-Hosseinabadi et al. [21]), including over two thousand rheumatoid arthritis cases and a comparable number of controls, identified a protective association between the TT genotype and the dominant model (TT + CT) of the FokI polymorphism in the general population. Active VDR signaling suppresses proinflammatory T cell subsets (Th1, Th17) and enhances regulatory T cell (Treg) function, which are key processes in maintaining immune tolerance and modulating autoimmune inflammation. Similarly, a meta-analysis of 11 studies reported a significant association between the FokI polymorphism and Hashimoto’s thyroiditis risk in the general population. The C allele of FokI, associated with a potentially more active form of the VDR in the dominant model, was linked to a higher risk of HT [105].

*VDR* and other vitamin D pathway gene polymorphisms influence the physiological effect of vitamin D supplementation and an individual’s responsiveness to it. This variability can lead to acquired vitamin D resistance, a complex phenomenon arising from interactions between genetic susceptibility and environmental factors. Individuals with such resistance may require very high, personalized doses of vitamin D to achieve adequate physiological and immunomodulatory effects. Low vitamin D status is a common phenomenon observed in patients with autoimmune diseases. Polymorphisms in vitamin D-metabolizing enzymes, such as *CYP2R1* (responsible for 25-hydroxylation) and *CYP27B1* (responsible for 1α-hydroxylation), also contribute to this resistance. For example, the rs7116978 CC genotype in *CYP2R1* has been identified as a strong predictor of low responsiveness to vitamin D supplementation. These variants influence both circulating vitamin D levels and susceptibility to autoimmune diseases such as type 1 diabetes and multiple sclerosis [36,107].

Specific VDR variants and supplementation outcomes:

- TaqI (rs731236): The GG genotype has been identified as a strong predictor of low vitamin D responsiveness in a supplementation study involving 100 Arab women. This variant affects VDR expression and has also been linked to increased risk of multiple sclerosis and rheumatoid arthritis [36,106,107],
- ApaI (rs7975232) and FokI: These polymorphisms influence mRNA stability and translation, thereby modulating serum vitamin D concentrations. Meta-analyses indicate that ApaI and FokI variants are associated with altered vitamin D status and susceptibility to vitiligo, supporting the hypothesis that these SNPs determine the serum vitamin D threshold required for immunological benefit [36,106].

## 7. Vitamin D Receptor mRNA Expression and Its Impact on the Development of Hashimoto’s Thyroiditis

The FokI (rs2228570) polymorphism of the *VDR* gene leads to the formation of two protein isoforms: F-VDR and f-VDR. The F-VDR isoform produces higher transcriptional activity dependent on NF-kB compared to f-VDR. In people with the FF VDR genotype, higher mRNA expression is observed in dendritic cells and monocytes compared with people with the ff *VDR* genotype, in whom this expression is lower. The type of genotype also affects the number of cytokines secreted. In the case of the FF genotype, IL-12 synthesis is higher than in the case of the ff genotype. These findings suggest that individuals with the FF genotype have a stronger immune response, which may increase their susceptibility to the development of autoimmune diseases like HT [1,20].

It has been emonstrated that inappropriate mRNA expression was observed in T lymphocytes from 45 patients with Hashimoto’s thyroiditis, resulting in reduced VDR transcription levels compared with 13 euthyroid controls. In summary, *VDR* gene polymorphisms—particularly FokI, BsmI, and TaqI—may alter *VDR* expression and downstream signaling, leading to dysregulated cytokine production and T-cell responses. These molecular alterations likely play a crucial role in the immunopathogenesis of autoimmune thyroid diseases by disturbing the delicate balance between pro- and anti-inflammatory mechanisms [109].

## 8. Conclusions and Outlooks

### 8.1. Future Directions

Based on the knowledge contained in this review, future directions may be identified, which may include areas such as:

1. Investigating the association between specific polymorphisms in vitamin D receptors and the response to vitamin D supplementation, including assessing the vitamin D dose–response relationship and the potential association with other genetic and environmental factors. Such findings could contribute to the development of personalized supplementation strategies.
2. Conducting in-depth mechanistic studies on the molecular pathways through which VDR and vitamin D influence immune cells, including cytokine production and T cell differentiation.
3. Conducting long-term clinical trials to assess the impact of vitamin D supplementation on the severity of Hashimoto’s thyroiditis and patient quality of life. Clinical symptoms of Hashimoto’s thyroiditis and autoantibody titers should be assessed. Such studies would establish evidence to support therapeutic recommendations.
4. Examining the influence of sex and age on VDR density, receptor functionality and immune responses occurring in Hashimoto’s thyroiditis. Such studies would aim to confirm the susceptibility of specific population types to the disease.
5. Investigating the impact of individual polymorphisms in genes encoding *VDR* on the risk of developing Hashimoto’s thyroiditis, potentially identifying groups of patients at risk for more severe disease and requiring early diagnosis.

This review explores the role of vitamin D deficiency and differences in vitamin D receptor density in the regulation of the immune system and immune responses in Hashimoto’s thyroiditis, considering future directions.

In conclusion, the VDR is essential for the activation of vitamin D, enabling it to fulfill its biological functions. The involvement of vitamin D in the immunological processes occurring in Hashimoto’s thyroiditis is extensive. Vitamin D deficiency results in the prevalence of processes that cause damage to thyroid cells and the development of Hashimoto’s thyroiditis. Recent findings indicate that vitamin D may have an immunomodulatory effect on HT by, among other things, influencing dendritic cell-dependent T-cell activation, downregulating HLA class II gene expression in the thyroid, preventing excessive B-cell response, and balancing the Th17/Treg cell ratio.

Vitamin D status and its bioavailability in the population are influenced by environmental and epigenetic factors, as well as the presence of various *VDR* gene polymorphisms.

These polymorphisms may have various effects depending on the region of the polymorphism. This determines the occurrence of various functional implications for the VDR protein and mRNA.

The type of polymorphism also influences the effectiveness of vitamin D supplementation, which is recommended due to the high prevalence of vitamin D deficiencies in the blood among the population. During supplementation, the concentration of vitamin D should be monitored due to the risk of side effects associated with over-supplementation, such as hypercalcemia or hypercalciuria.

Although the effect of vitamin D on the proper functioning of the thyroid gland has not been fully studied, numerous studies have shown that its concentration in the blood maintained within the age-appropriate range of the norm may act preventively in autoimmune diseases. Further well-designed findings should be carried out to improve understanding of the role of *VDR* gene polymorphisms in the modulation of the response to vitamin D supplementation, and its possible clinical value. However, current findings regarding its role in Hashimoto’s thyroiditis remain inconsistent and often vary between populations, highlighting the need for further investigation to clarify these associations.

### 8.2. Conclusions

Based on the literature review, it appears that *VDR* gene polymorphisms may alter both the structure and function of the receptor, and specific polymorphisms may influence how the body utilizes available vitamin D. Therefore, individual differences in response to vitamin D may occur. Relatively stronger evidence pertains to the FokI and TaqI polymorphisms. Most meta-analyses and case–control studies have shown that these polymorphisms are either not associated with HT or exhibit a statistically significant protective effect that reduces the risk of its development. The presence of FokI and TaqI polymorphisms is also associated with improved effectiveness of vitamin D supplementation. However, it is important to note that the effect of polymorphisms is population-specific and has not been observed in every studied population. Furthermore, most studies focus on the association of VDR polymorphisms with susceptibility to developing Hashimoto’s thyroiditis, not directly on the severity of disease symptoms. It should be noted that the number of studies is relatively limited and typically involves case–control studies analyzing polymorphisms in groups of 70–200 patients from a specific population. Larger multi-ethnic cohort studies and standardized genotyping protocols are clearly needed as precise directions for future research in order to draw consistent conclusions, determine cause-and-effect relationships, and establish potential clinical significance. Moreover, research should also focus on the regulation of *VDR* expression; perhaps epigenetic factors, which are a specific link between genes and the environment, the effect of which is modified by the unique genetic and environmental background characteristic of a given population, can effectively modulate the influence of polymorphism, thereby determining gene activity.

## Figures and Tables

**Table 1:** The most important VDR polymorphisms and their impact on receptor VDR protein function or density [27].

VDR Polymorphism Region	Polymorphism Name	Effect	Result
exon	FokI (rs10735810/rs2228570)	production of a shortened protein	increased transcriptional activity of the protein
exon	TaqI (rs731236)	affects *VDR* gene methylation status	altered gene transcription
intron	ApaI (rs7975232)	affects transcript stability	altered VDR protein translation and density status
intron	BsmI (rs1544410)	affects transcript stability	altered VDR protein translation and density status
promoter	GATA (rs4516035)	disruption of the binding to the GATA binding site	reduced VDR promoter activity
promoter	Cdx2 (rs11568820)	changes overall activity and VDR transcription	altered active (bonded to VDR) vitamin D activity

Polymorphisms: BsmI (rs1544410), ApaI (rs7975232) and TaqI (rs731236) are typically considered “silent” genetic variants, meaning they do not cause changes in the amino acid sequence of the encoded VDR protein [27].

## Data Availability

No new data were created or analyzed in this study. Data sharing is not applicable to this article.

## Supplementary Materials

The following supporting information can be downloaded at: https://www.mdpi.com/article/10.3390/ijms262110576/s1.
