# Supplementary material for: Vitamin D Receptor Polymorphisms and Immunological Effects of Vitamin D in Hashimoto’s Thyroiditis
Source: Int J Mol Sci. 2025 Oct 30;26(21):10576. doi: 10.3390/ijms262110576 (PMC12609546; doi:10.3390/ijms262110576)
Supplement: Supplementary file 1 [file ijms-26-10576-s001.zip › ijms-3909081-supplementary.pdf]

Supplementary Materials

Table S1. Comparative table of the characteristics of individual studies on the impact of VDR polymorphisms and serum 25(OH)D levels in relation to various diseases.

Author and Year of Investigation	Population Backgrounds	Sample Size By Case Group	The Disease Under Investigation	Type of Investigation	Results and Outcomes
Ma et al., 2020 [49]	Chinese	674 participants:<br>-1st group -<br>138 participants, including 76 femalesand 62 males (mean, 60.3 ± 2.3 years)<br>-2nd group -<br>536 participants, including 279 females and 257 males (mean, 63.6 ± 1.2 years).	All participants have diabetes type 2:<br>-1st group – with CAD[1],<br>-2nd group – without CAD	cross-sectional study	1. Type 2 diabetes patients with CAD had the lowest vitamin D levels.<br>2. FokI and TaqI polymorphisms were significantly more common in type 2 diabetes patients than in the control group.
Suksawatamnuay et al., 2020 [50]	Thai	182 participants, including 79 females and 103 males (mean, 62 ± 11 years)	Colorectal cancer	case-control study	There were no significant differences in allele and genotype frequencies for the VDR polymorphisms: FokI, BsmI, Tru9I, ApaI and TaqI between the case group and the control group.
Iriani et al., 2024 [51]	Indonesian	45 participants, including 17 females and 28 males aged over 18 years old	Lymphoma or leukemia	cross-sectional study	The level of VDR protein was found to be significantly higher in males than in females.
Kazemian et al., 2022 [57]	Patients admitted to a hospital in Iran	214 women aged 25-65 years	Breast cancer survivors	single-arm non-randomized pre- and post trial	1. In women with the tt TaqI genotype, there was a greater decrease in plasma matrix metallopeptidase 9 levels compared to the TT genotype after vitamin D3 supplementation.<br>2. VDR TaqI and BsmI sequence variants modified the effect of vitamin D3 treatment on changes 25(OH)D in plasma.
Al-Ghafari et al., 2019 [58]	Saudi	50 participants, including 12 females and 38 males aged 30-80 years	Colorectal cancer	case-control study	1. For the ApaI polymorphism, a significantly higher serum total vitamin D level was observed only in colorectal cancer patients with the homozygous aa genotype, compared to those with the heterozygous Aa or homozygous dominant AA

					genotypes.<br>2. Colorectal cancer patients with the heterozygous Tt or homozygous recessive tt genotype of the TaqI polymorphism exhibited lower serum total vitamin D concentrations compared to patients with the homozygous dominant TT genotype.<br>3. The analysis did not reveal any statistically significant associations between the genotypes of the BsmI and FokI polymorphisms and total vitamin D levels.
Latacz et al., 2021 [59]	Caucasian	103 participants, including 42 females and 61 males aged 57-82 years	Colorectal cancer	cross-sectional study	1. After adjusting for confounding factors in logistic regression analysis, none of the TaqI and BsmI genotypes or alleles demonstrated a statistically significant association with colorectal cancer susceptibility.<br>2. For the ApaI and FokI polymorphisms, no correlation was observed between genotypes or alleles and susceptibility to colorectal cancer.
Kumar et al., 2020 [60]	Indian	75 participants, including 21 females and 54 males aged 38-78 years	Newly diagnosed multiple myeloma	case-control study	1. Patients with multiple myeloma showed significantly reduced levels of 25(OH)D compared to the control group.<br>2. A significant association was observed between the occurrence of multiple myeloma and the presence of the *f* allele of the FokI polymorphism, the *a* allele of the ApaI polymorphism, and the *b* allele of the BsmI polymorphism.
Rui et al., 2019 [61]	Patients admitted to a hospital in Fujian Province (China)	40 participants, including 17 females and 23 males aged 34-81 years	Multiple myeloma	case-control study	1. The A allele of the BsmI polymorphism may be strongly associated with the development of multiple myeloma.<br>2. The C allele of the TaqI polymorphism shows a positive association with the development of multiple

					myeloma.
Hassan et al., 2021 [62]	Patients admitted to a hospital in Egypt	105 participants, including 77 females and 28 males (mean, 45.4 ± 11.5 years)	Knee osteoarthritis in various degrees of advancement	case-control study	1. Patients in the case group exhibited significantly lower serum 25(OH)D levels compared to the control group. The 25(OH)D level did not differ according to disease severity.<br>2. The GG wild-type and GT heterozygous genotypes of the ApaI polymorphism were more frequently observed in patients with knee osteoarthritis than in the control group.<br>3. The CC genotype of the TaqI polymorphism increases the risk of developing knee osteoarthritis, whereas the TT and TC genotypes may have a protective effect.
Shokri et al., 2021 [63]	Egyptian	20 female participants (mean, 24.4 ± 8.274 years)	Osteomalacic myopathy	case-control study	1. Female patients in the case group had significantly lower vitamin D levels compared to those in the control group.<br>2. The FokI polymorphism of the VDR gene significantly increases the risk of osteomalacic myopathy, but does not affect the severity of the clinical course, similarly to the ApaI and BsmI polymorphisms.
Marini et al., 2020 [40]	Italian	103 participants who were children, adolescents, and young adults, including 82 females and 21 males (mean, 20.21 ± 7.11 years)	Juvenile idiopathic arthritis	cross-sectional study with case-control component	1. The vast majority of patients with juvenile idiopathic arthritis were found to have vitamin D deficiency or insufficiency.<br>2. The TT genotype of the ApaI polymorphism was significantly more frequent in patients with juvenile idiopathic arthritis, whereas the GT and GG genotypes were markedly less common in these patients compared to the control group.<br>3. No significant differences were observed in the distribution of BsmI, Cdx2, FokI, and TaqI genotypes between patients with juvenile idiopathic arthritis and the

					control group.
Lye et al., 2020 [64]	Malay, Chinese, Indian and other	300 participants aged 18–65 years, including 200 females and 100 males	Single or recurrent non-psychotic episode of major depressive disorder diagnosed less than 2 years before enrolment in the study	case-control study	No significant association was found between the BsmI, ApaI, and TaqI polymorphisms and the development of major depressive disorder.
Zhang et al., 2021 [65]	Chinese	1120 participants, including 172 females and 948 males aged over 18 years old (mean, 40.48 ± 8.80 years)	Non-alcoholic fatty liver disease	case-control study	1. The incidence of non-alcoholic fatty liver disease was significantly higher in patients with vitamin D deficiency compared to patients with vitamin D insufficiency or sufficiency.<br>2. High serum VDR levels and VDR polymorphism variants (rs2228570-A and rs11168287-A) may contribute to the low risk of non-alcoholic fatty liver disease.
Qadir et al., 2021 [66]	Patients admitted to a hospital in Srinagar (India)	143 participants (mean, 53.5 ± 7.92 years). No data regarding gender distribution.	Newly diagnosed gastric cancer	case-control study	1. The GA/AA genotypes of the BsmI polymorphism were significantly associated with an increased risk of gastric cancer development compared to the wild-type GG genotype, both in the case group and the control group.<br>2. No significant association was observed between the ApaI and TaqI polymorphisms and the development of gastric cancer.<br>3. The BsmI, ApaI, and TaqI SNPs[2] were significantly associated with reduced overall survival in gastric cancer patients.
Yang et al., 2022 [67]	Patients admitted to a hospital in Yunnan Province (China)	576 participants (mean, 53.9 ± 12.7 years). No data regarding gender distribution.	Sepsis developed in the last 24 hours	case-control study	1. Patients with sepsis exhibited lower vitamin D levels compared to healthy individuals in the control group.<br>2. The presence of the FokI polymorphism predisposes to the development of sepsis. The *f* allele of FokI was more frequently observed in patients with sepsis than in the control group.<br>3. The distribution of alleles

					and genotypes of the TaqI, BsmI, and ApaI polymorphisms was similar between the patient group and the control group.
Kim et al., 2022 [68]	Korean	172 participants, including 103 females and 69 males aged 3-85 years (mean, 40.96 ± 22.22 years)	Vitiligo	case-control study	1. The mean serum 25(OH)D level in the case group did not differ significantly from that in the healthy Korean population.<br>2. No significant differences in VDR polymorphisms were found between the case group and the healthy control population.
Jafari et al., 2021 [69]	Iranian	120 infertile women aged 19-45 years (mean, 32 ± 12 years)	Endometriosis	case-control study	1. There were no significant differences in the genotype distribution and allele frequencies of VDR polymorphisms (FokI, BsmI, TaqI and ApaI) between patients with endometriosis and the control group.
Aziz et al., 2023 [70]	Pakistani	40 pregnant women during their third trimester of pregnancy (mean, 33.05 ± 5.26 years)	Preeclampsia	case-control study	1. It was shown that neither the 25(OH)D level nor the ApaI polymorphism was significantly associated with the incidence of preeclampsia.
Punceviciene et al., 2021 [71]	Lithuanian	206 participants, including 184 females and 22 males (mean, 55.01 ± 11.08 years)	Rheumatoid arthritis	case-control study	1. In patients in the case group, the mean serum 25(OH)D level was significantly lower compared to the control group.<br>2. No association was found between any allele or genotype of the VDR polymorphisms (FokI, BsmI, TaqI, and ApaI) and the risk of developing rhematoid arthritis.<br>3. No significant differences were found in the distribution of VDR polymorphism genotypes between the case and control groups.

[1]CAD: coronary artery disease; [2]SNPs: single nucleotide polymorphisms

Overview of study results by population ethnicity and sample size

Studies in Table S1. with large or moderate sample sizes that showed an association include Ma et al. [49] (674 participants), Kazemian et al. [57] (214 participants), Zhang et al. [65] (1120 participants), Yang et al. [67] (576 participants). Significant associations were also found in studies with fewer than 200 participants: Al-Ghafari et al. [58] (50 participants), Kumar et al. [60] (75 participants), Rui et al. [61] (40 participants), Hassan et al. [62] (105 participants), Shokri et al. [63] (20 participants), Qadir et al. [66] (143 participants) and Aziz et al. [70] (40 participants). Most participants in these studies were from Asia.

No significant associations were observed in both moderate and small sample sizes. This applies to the studies: Suksawatamnuay et al. [50] (182 participants), Latacz et al. [59] (103 participants), Marini et al. [40] (103 participants), Lye et al. [64] (300 participants), Kim et al. [68] (172 participants), Jafari et al. [69] (120 participants), and Punceviciene et al. [71] (206 participants). In most cases, participants were from Asia.
